# Arnica as a Remedy for Boils

**Published:** 1878-05

**Authors:** 


ARNICA AS A REMEDY FOR BOILS.—In the *Journal de Therapeutique* for January 25, 1878, Dr. Planat writes that he has found arnica possessed of rapid and constant efficacy in case of boils. He was led to try arnica in these cases from the result of physiological experiments made by him, with a view of studying the *modus operandi* of this substance on wounds. Its property of producing resolution, evidently due to its influence on the vaso-constrictor nerves, gave him the idea of applying it in all cases of superficial inflammation, such as boils, angina, erysipelas, etc. These experiments have convinced M. Planat that arnica arrests all furuncular eruptions with remarkable rapidity. M. Planat makes an exception in the case of diabetic boils, which have not come under his observation, and of carbuncle, which, by reason of its exceptionally serious character, he has treated in the ordinary way. He has been equally successful in cases of erysipelas and acute simple angina, but is not quite so clear about this as of the case of boils. The arnica was applied directly to the inflamed parts in the form of an ointment, composed of 10 grammes of extract of fresh arnica flowers to 20 grammes of honey. If this mixture be too thin, lycopodium or althea powder, or any similar substance, may be added so as to give it the necessary consistence. It is spread on diachlon plaster or oiled silk, and applied to the boil. Generally it is sufficient to renew this dressing once in twenty-four hours. Two or three applications generally cause the boil to die away at all stages of its evolution.

Dr. Planat has also given internally, in cases of this character, tincture of arnica in doses of from 25 to 30 drops in a draught to be taken in teaspoonfuls every two hours, and has thereby obtained so rapid an extinction of the furuncular eruption that it seemed impossible to him to deny the special action of the drug. He, however, noted greater efficiency from its direct application.—*Lond. Rec. February* 15, 1878.

